# Genome Sequences of Two Strains of *Salmonella enterica* Serovar Enteritidis Isolated from Shell Eggs

**DOI:** 10.1128/genomeA.00954-15

**Published:** 2015-09-10

**Authors:** Matthew R. Moreau, Dona Saumya S. Wijetunge, Eranda Mangala K. Kurundu Hewage, Bhushan M. Jayarao, Subhashinie Kariyawasam

**Affiliations:** a Department of Veterinary and Biomedical Sciences, The Pennsylvania State University, University Park, Pennsylvania, USA; b Department of Food Science, The Pennsylvania State University, University Park, Pennsylvania, USA; c Animal Diagnostic Laboratory, The Pennsylvania State University, University Park, Pennsylvania, USA

## Abstract

This report presents the complete genome sequences of two *Salmonella enterica* serovar Enteritidis strains bearing the pulsed-field gel electrophoresis profile JEGX01.0004, which were isolated from the internal contents of eggs.

## GENOME ANNOUNCEMENT

*Salmonella enterica* serovar Enteritidis (SE) is one of the most common causes of bacterial foodborne illness in humans (1). Over the last two decades, cases of SE have increased in humans by approximately 44% (2). *S. enterica* serovar Enteritidis can infect a wide range of hosts by use of many virulence factors, including those found within five major *Salmonella* pathogenicity islands (3–5). Though most people develop gastroenteritis due to SE, some individuals may get systemic infections (6). Among various sources of SE, eggs and egg products are the primary vehicles for most SE outbreaks (2).

Hens can be colonized with SE asymptomatically through the fecal-oral route from hens or rodents that are infected and shed the bacteria in feces or by vertical transmission from the breeder chicken to the fertile egg (7, 8). Once internalized, SE colonizes various parts of the gastrointestinal and reproductive tracts; both of which can lead to contamination of an egg at various points during its formation (9). One in every 20,000 eggs is estimated to be contaminated with SE, and with over 65 billion eggs produced, there are 3.25 million eggs contaminated each year in the United States alone (10). Here, we report whole-genome sequences of two SE strains (SEE1 and SEE2) isolated from shell eggs and belonging to the pulsotype JEGX01.0004, which is one of the most common PFGE fingerprint patterns of SE reported to PulseNet (11, 12).

Genomic DNA was isolated from overnight cultures with the Promega Genomic Wizard Kit (Promega Corp., Madison, WI) and sequenced using the Ion Torrent PGM sequencer (Life Technologies, Grand Island, NY) at the Genomics Core Facility of the Pennsylvania State University (University Park, PA), which generated 1,042,678 reads (approximately 85× coverage of the genome) for the SEE1 genome and 2,885,240 reads (approximately 116× coverage of the genome) for the SEE2 genome. Both chromosomes were subjected to optical mapping (OpGen, Inc., Gaithersburg, MD) and visualized using MapSolver software version 3.0 (OpGen, Inc., Gaithersburg, MD). Using a combination of SeqMan NGen Software v11 (DNASTAR, Madison, WI) and the optical map, the reads assembled by a reference guided assembly with the P125109 genome (GenBank accession number AM933172.1). The one major gap was found to match a portion of the genome of *Salmonella* Gallinarum strain 287/91 (GenBank accession number AM933173.1), which closed the gap.

All remaining gaps were closed by extension of the short reads. Once the optical maps matched and the genomes circularized, the genomes were considered closed. Both genomes are approximately 4.67 Mb long with ~4,600 open reading frames (approximately 1 gene/Kb). There are 22 rRNA genes and 84 tRNA genes and, as a whole, the G+C content of each genome is approximately 52%. The information generated from the genomes of egg origin SE will play an important role in further studies to increase our understanding of the genetic landscape of SE during egg contamination prior to human consumption.

### Nucleotide sequence accession numbers. 

Genome sequences of SEE1 and SEE2, along with genome annotations, have been deposited in the GenBank database (NCBI) with the accession numbers CP011790 (SEE1) and CP011791 (SEE2).
